# From Mechanical Machining Technology: A New Solution That Integrates Blades to the Implant to Control the Stress to the Peri-Implant Cortical Bone

**DOI:** 10.3390/bioengineering11111077

**Published:** 2024-10-28

**Authors:** Mauro Ferri, Marco Guzzo, Hiroyuki Omori, Yuma Hazama, Nicodemo Vittorio Masotta, Daniele Botticelli

**Affiliations:** 1 Private Practice, Cartagena de Indias 130001, Colombia; medicina2000ctg@hotmail.com; 2 Brenta Engineering, 35027 Noventa Padovana, PD, Italy; marco.guzzo@brentaengineering.it; 3 Department of Oral Implantology, School of Dentistry, Osaka Dental University, 8-1 Kuzuhahanazonocho, Hirakata 573-1121, Osaka, Japan; last_samurai_1206@me.com (H.O.); y.hazama.d@gmail.com (Y.H.); 4 Private Practice, 88074 Crotone, CA, Italy; nicodemo.masotta@gmail.com; 5 ARDEC Academy, 47923 Rimini, EM, Italy

**Keywords:** bone loss, cortical decompression, peri-implant resorption, osseointegration with autologous bone, alveolar precision

## Abstract

Background: To prevent excessive compression of the cortical layer, which can lead to marginal bone loss, various companies have introduced specialized drills. However, these drills often lack the necessary precision, as the operator’s hand may neither be stable enough to prevent ovalization and over-widening nor precise enough to maintain coaxial alignment. Therefore, the aim of this study was to develop a device capable of achieving calibrated cortical preparation in terms of both dimension and coaxiality. Methods: A machining technology based on drilling principles was employed to create the device. Results: Nine blades were incorporated between the transmucosal neck and the implant threads, enabling the blades to cut the cortical bone coaxially during the implant insertion process. Conclusions: The primary goal of this study was to develop an implant capable of achieving calibrated cortical bone preparation, ensuring both precise dimensional control and coaxial alignment. This design incorporates integrated blades that allow for controlled cortical decompression, helping to manage radial compressive stresses during implant placement. Although the experimental studies cited were conducted independently of this research, they validate the functional efficacy of this implant design, demonstrating its ability to promote osseointegration and preserve marginal bone. The results suggest that this implant configuration holds the potential for improving clinical outcomes, particularly in cases where bone quality or density poses challenges to implant stability.

## 1. Introduction

One of the preliminary operations that must be performed for the installation of a dental implant in the alveolar bone is to properly prepare for osteotomy of the recipient sites using special tools. The most common osteotomy preparation system uses drill bits. However, ultrasonic devices have been successfully applied for implant site preparation [1,2,3]. Animal studies have demonstrated comparable outcomes in terms of bone levels and osseointegration for implants placed in osteotomies prepared with either drills or ultrasonic instruments [4,5]. A randomized clinical trial [6] and a prospective clinical study [7] confirmed these findings despite the longer preparation time required for ultrasonic devices compared to drills [6,7]. Sonic instruments have also been used for implant site preparation in both animal [8] and human studies [9], showing favorable healing and integration of the implants. The healing of implants placed in sites prepared with either drills or sonic devices has been evaluated in both animal [10] and human [11] studies, with similar osseointegration results observed.

The customized, large-scale production of cutting-edge implants represents a significant application within the dental field, including additive manufacturing [12]. Implants have also been designed to compact bone debris around them, aiming to promote better healing and osseointegration [13]. While the depth of the osteotomy preparation is adjusted to the length of the implant, the diameter is based not only on that of the implant but also on the quality of the alveolar bone, which may have different hardnesses [1,14,15,16,17]. Most implants on the market feature threads, and the osteotomy is typically prepared with a diameter smaller than the implant (including the threads) to achieve optimal primary stability [18,19,20]. Implant site preparation techniques, such as osseodensification [21] or implants with varying tapered designs [22] and thread profiles [18,19,20], also influence insertion torque. However, it should be noted that while preparing the osteotomy with a smaller diameter drill enhances implant stability, it may also induce microstrains and microcracks in the cortical bone surrounding the threads [23]. Additionally, research has shown that optimal osseointegration is achieved with insertion torque values between 30 and 50 Ncm compared to very low or excessively high values [24].

To avoid damaging compression of the alveolar bone, the clinician calibrates the diameter of the osteotomy to the hardness of the bone; that is, the harder the bone, the larger the diameter. Animal studies have suggested that high insertion torque may lead to marginal bone loss [25,26], whereas human studies have reported minimal marginal bone loss [27] and comparable osseointegration with implants installed with a standard torque [28]. Several reviews have found no association between insertion torque values and peri-implant marginal bone resorption [29,30,31].

To prevent such issues, various implant systems have introduced specialized drills designed to expand the cortical area of the osteotomy. However, performing this step manually does not ensure the required accuracy, as the operator’s hand may lack the necessary steadiness to prevent ovalization or excessive widening and may not maintain perfect coaxial alignment.

Moreover, during installation, the implant often follows the path of least resistance through the bone, potentially deviating from the intended trajectory, even if the operator tries to adjust during the threading process. Hence, the aim of this study was to develop a device capable of achieving calibrated cortical preparation in terms of both dimension and coaxiality.

## 2. Founding Paradigms

For this purpose, blades were made between the thread and transcortical collar of the implants.

**Figure 1:**
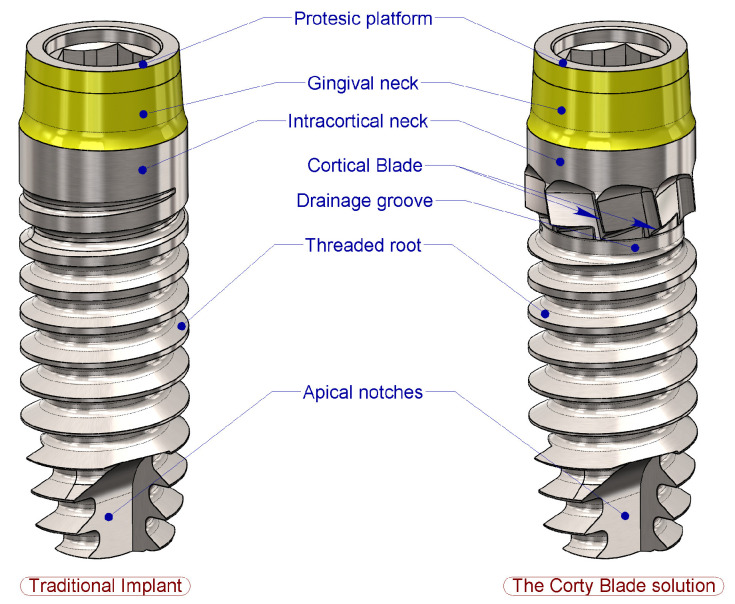
Comparison of a traditional solution and the innovative implant configuration. The Corty blade solution consisted of inserting cortical blades and a drainage groove between the intercortical collar and the end of the threaded root in coronal region.

Such blades can widen the cortical layer during implant insertion at a calibrated diameter and depth with perfect coaxiality with the implant (Figure 1).

Since the blades are designed to expand a pre-existing cylindrical osteotomy, the functional mechanism of these blades relies on drilling technology. To achieve the intended cutting action, it is necessary to employ more than one blade, especially when multiple implant revolutions are required. This is because using only one blade per revolution would result in excessive depth penetration, leading to the formation of large bone chips and increasing the risk of the implant becoming stuck upon entering the cortical bone.

**Figure 2:**
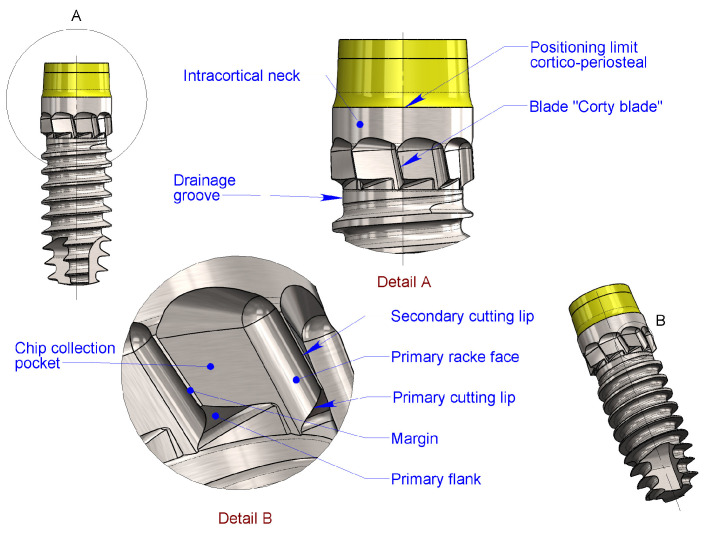
Detail of blades and drainage groove—parts nomenclature. In Detail A, the implant positioning referring to the bone is shown. In Detail B, the nomenclature of principal single-blade parts is described.

For optimal performance of these blades—allowing them to effectively engage the cortical bone in the axial direction during screwing—a specially designed drainage groove must be created. This groove, positioned between the coronal thread and the cortical blades, provides sufficient space for the cutting edges of the blades to function efficiently (Figure 2).

The blades can operate with varying diameters, depending on the design. The difference between the diameter of the hole created by the blades and the diameter of the trans-cortical collar is referred to as the Blade Diameter Differential (BDD).

**Figure 3:**
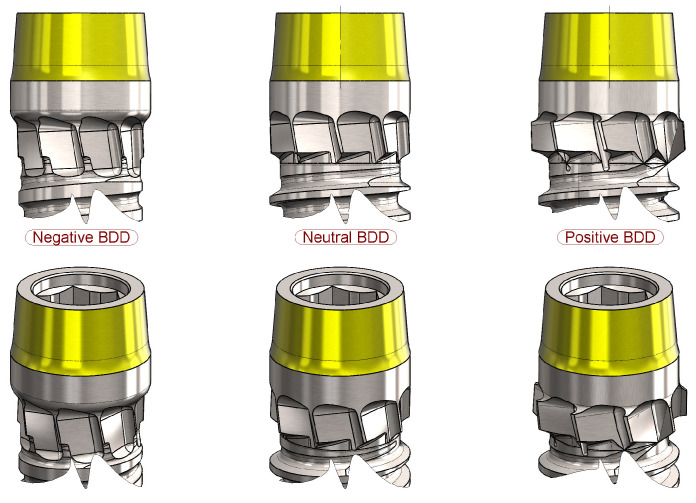
Schematic representation of BDD differential type. Highlight—Negative BDD: The external diameter of the blade (d3) is smaller than the intracortical neck diameter (d4). Neutral BDD: The external diameter of the blade (d3) is equal to the intracortical neck diameter (d4). Positive BDD: The external diameter of the blade (d3) is greater than the intracortical neck diameter (d4).

The BDD is classified as neutral when it matches the diameter of the trans-cortical cylinder, producing no compressive or decompressive effect. A positive BDD occurs when a circumferential marginal space is formed between the cortical bone and the trans-cortical cylinder, leading to a decompressive effect. Conversely, a negative BDD is observed when the maximum blade diameter is smaller than the trans-cortical cylinder diameter, creating a controlled interference with the cortical bone layer and resulting in a compressive effect (Figure 3).

In any case, controlled decompression/compression is focused exclusively on the trans-cortical cylindrical portion, achieved through a mechanical removal process carried out by the blades after the implant thread has been fully inserted into the cortical bone.

As a result, the compression exerted by the thread during the intermediate screwing stages is maintained. However, due to the subsequent action of the blades and the relief groove positioned beneath them, this compression becomes transient, applied only for the brief time required for the thread ridges to pass through the cortical bone, concluding their path in the medullary bone.

**Figure 4:**
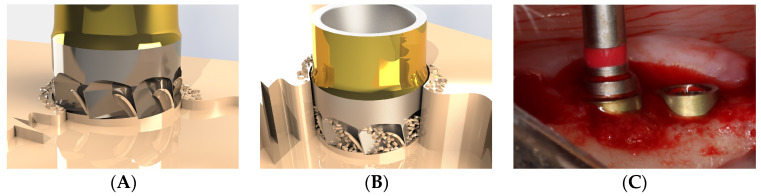
Conceptualization of the blade effect at end of implant placement. (**A**) First contact of the blades with the cortical bone. (**B**) Bone chips ejected outside and harvested within the collecting pockets. (**C**) Excess of autologous material ejected from the blade cavities in vivo (dog).

The flutes (grooves) designed to create the cutting edges of the blades serve to initially expel part of the bone chips into the immediate peri-implant area. As the blades progressively penetrate the cortical bone, these chips are forced into the prepared osteotomy. Once this space is filled, and before the transcortical collar acts as a plug, the flutes expel any excess chips toward the external surface, ultimately contributing to bone regeneration (Figure 4A–C).

### 2.1. Description of the Implant^®^

**Figure 5:**
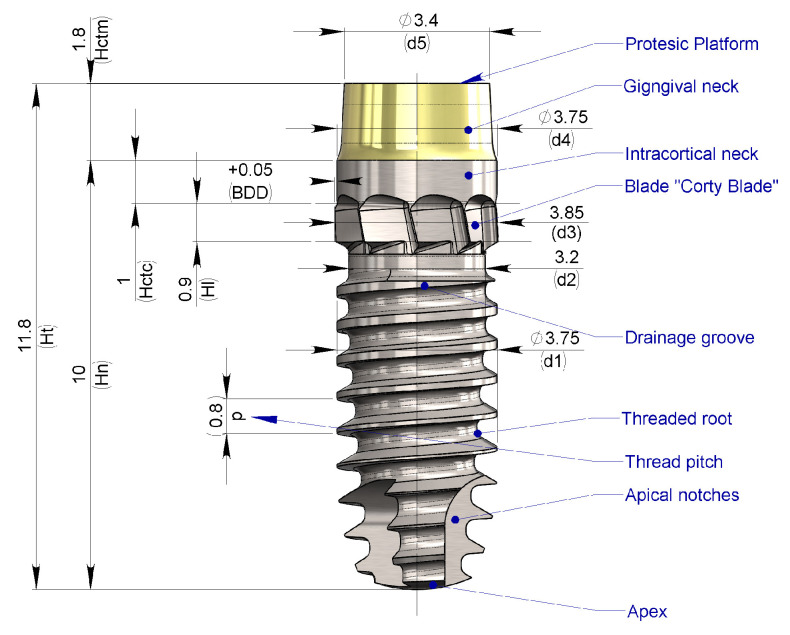
Dimension nomenclature—Reference drawing—(d1) Nominal implant diameter—(d2) Drain groove diameter—(d3) Blade external diameter—(d4) Intracortical neck diameter—(d5) Prosthetic platform diameter—(Hn) implant nominal height—(Hctm) Transmucosal neck height—(Hctc)—Intracortical neck height—(Hl) blade height—(BDD) Blade differential diameter—(p) Thread pitch.

The reference dimensions and corresponding nomenclature are illustrated in Figure 5. The implant used as a reference example (CortyBlade^®^ Leader Medica s.r.l., via Giacinto Longhin 11, Padova, Italy) has the following key specifications: a nominal length (Hn) of 10 mm; a nominal external thread diameter (d1) of 3.75 mm; a transmucosal neck height (Hctm) of 1.8 mm; a transcortical collar diameter (d4) of 3.75 mm; an external blade diameter (d3) of 3.85 mm; and a prosthetic platform diameter (d5) of 3.4 mm.

### 2.2. Mechanics of Bone Chip Formation

**Figure 6:**
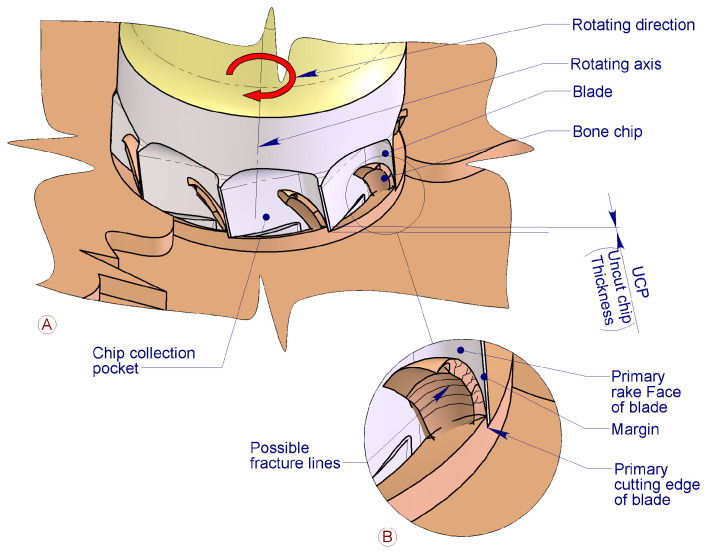
The figure illustrates the chip formation by the corty blades, defining component’s name of the cortical blades that contribute to its generation. (**A**), overview of all blades in action; (**B**), detail of a single blade.

**Figure 7:**
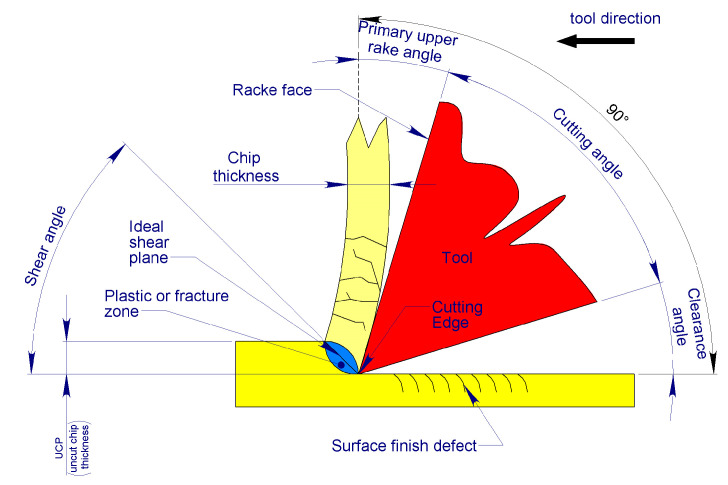
The figure illustrates the mechanism of bone chip formation executed by the cortical blades, highlighting the areas of the blades and the bone involved in the processing.

Bone chips are generated during the screwing process of the implant by the primary cutting edge located at the lower end of the wedge-shaped blades, which constitute the main component responsible for performing the osteotomy (Figure 6 and Figure 7).

The wedge of the primary cutting edge, rotating in unison with the tool, penetrates the material through indentation. This process is facilitated by the action of the cutting edge, which forms an inclined surface relative to the working direction, known as the rake face of the tool. The penetration of the cutting edge leads to the separation of material through plastic deformation or fracture, causing the formation of a thin layer that subsequently slides over adjacent material layers. These layers, while retaining a degree of residual ductility, accumulate to form an interconnected and continuous multilayer “sandwich”. As these layers slide across the rake face of the tool, they form bone chips. This mechanism aligns closely with Merchant’s model for isotropic materials.

It is important to consider that cortical bone, the primary target for blade processing, is a distinctly anisotropic material composed of hierarchical structures [32]. Its resistance to cutting varies significantly depending on whether the processing occurs along the transverse, parallel, or normal directions relative to the osteons [33,34,35].

As a result, Merchant’s model [36], which assumes the material is perfectly isotropic, presents limitations in accurately representing the mechanism of chip formation in cortical bone.

**Figure 8:**
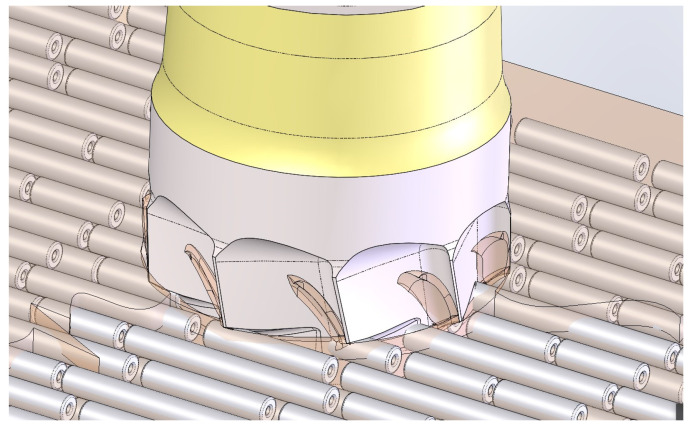
Conceptualization of the distribution and orientation of osteons with respect to the placement of the implant.

Nomura et al. [37] determined that the orientation of osteons in the human mandible aligns with the main axis characteristic of a “horseshoe”-shaped bone. When applying these findings to dental implant procedures, it becomes evident that the direction in which the blades encounter the osteons varies continuously (Figure 8). This variation is largely due to the circular motion of the blades. A similar observation was made by Zhang et al. [38] in their studies on advanced machining of hard tissue.

Although cutting procedures generally occur in multiple directions, Merchant’s model [36] can still serve as a valuable reference for the design of tools intended for bone processing [33].

As a result, bone chips formed during machining can exhibit different structural conformations. Liao et al. [39] identified three distinct chip formation mechanisms: shear cutting; mixed shear/fracture cutting; and fracture cutting. Interestingly, the structure of the bone chip is largely independent of the shear direction relative to the osteon orientation, but it is more influenced by the depth of the pass.

Liao et al. [39] demonstrated that, regardless of osteon orientation, (i) an Uncut Chip Thickness (UCT) of 0 to 20 µm produces a continuous, flowing chip (shear cutting mechanism) in bovine cortical bone, which may also curl; (ii) a UCT between 20 and 80 µm results in a continuous, segmented chip (mixed shear/fracture cutting mechanism), and (iii) a UCT of 80 to 150 µm leads to the formation of discontinuous chips (fracture cutting mechanism), which fracture coarsely. However, their experiments were conducted on non-viable cortical bone samples from femurs, which lacked the natural hydration provided in vivo by the Haversian system.

In contrast, Robles-Linares et al. [40] investigated cutting conditions using fresh, non-viable bovine bone, artificially hydrating the Haversian system with saline solution to more closely simulate natural tissue hydration. Under these conditions, with the same UCT, they observed similar chip formation, as reported by Liao et al. [39]. However, the hydrating effect of the saline in the cutting zone caused the chip to remain parallel to the cutting surface (tool chest) rather than curling. Additionally, the presence of fluid in the Haversian canals led to superior surface finishing, leaving the canals and canaliculi open and free of obstructions caused by material crushing or tearing [40].

### 2.3. Defining the Type of Swarf to Be Produced by the Blades

For the application presented in this study, a continuous and flowing chip, particularly one that may curl, should be avoided as it can clog the blade collection grooves by entangling [41], forming a large mass, generating excessive friction against the walls of the implant osteotomy, and exerting significant pressure on the freshly worked bone, potentially leading to damage.

A coarse, discontinuous chip, primarily formed through fracture, can cause issues similar to flowing chips. Moreover, since these chips are formed by fracture, they may induce microcracks in the freshly machined surface, which could propagate into deeper fractures in the bone over time. Additionally, chip formation through fracture is associated with surface roughness [39], which can cause greater damage to the cut area.

In contrast, a continuous segmented chip would be ideal for the application in this study. First, because the surface finish is relatively smooth [39], it only affects small, shallow portions of the bone. Second, its predetermined, small segmentation lines allow it to easily break down into tiny chips, making it better suited for filling the blade grooves without causing further damage to the newly machined bone walls. These small chips can more easily conform to the collection spaces around the blades. Therefore, the continuous segmented chip type should be adopted as the operational basis for the blades.

## 3. The Solution^®^ in Practice

### 3.1. Parameters at the Main Cutting Edge

#### 3.1.1. Definition of Blade Vertex Angle

**Figure 9:**
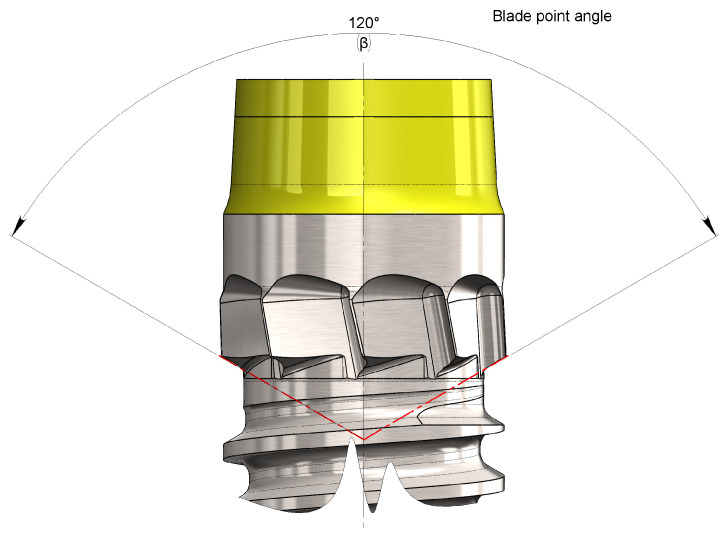
Representation of the apex angle of the cortical blade. Note how it resembles the vertex angle in the cutting apex of a traditional drill bit.

The angle at the vertex of the blades is denoted as β (Figure 9). This angle, as with a drill bit, influences numerous machining parameters, such as the following:

1. Axial penetration thrust: The smaller the angle, the lower the thrust required for the blade to penetrate the bone [34];
2. Self-alignment capability: The greater the angle, the lower the alignment capability [42];
3. Chip width: The smaller the angle, the larger the normal cross-section of the chip produced [42];
4. Chip thickness: The smaller the angle, the smaller the UCT thickness of the chip.
5. The quantity of heat generated is as follows: the wider the angle, the greater the creep deformation of the materials, and thus, the greater the heat produced [42].

The most desirable characteristic of blades is to maintain a good degree of self-alignment with the screw direction of the implant, preventing external forces, such as the thrust exerted by the operator, to deviate from the desired direction. To this end, a vertex angle β tending toward 90° has been suggested [43].

At the same time, however, there is a need to keep the screwing torque of the implant as low as possible to facilitate insertion by the operator working in confined spaces. In fact, the lower the applied external stress, the better the desired trajectory maintained.

Considering the limited extension of the main cutting edge of the blades, compared with a hypothetical conventional drill bit operating with the same diameter, the penetration thrust can be considered negligible. However, the resistance torque acting near the outer diameter of the blades remained relatively high. To limit the drilling torque, the use of a vertex angle β between 110° and 140° has been suggested [42].

The increased heat produced by using any tip angle can be considered negligible in this case, as the rotation speed of the blades is very limited because the system is expected to be tightened either with a contra-angle handpiece, setting it at a low rotation speed (≤20 rpm), or manually with a ratchet.

Considering the standardized dimensions of the tools available on the market for blade manufacturing, a β vertex angle of 120° was chosen. This decision was made because it was considered that penetration forces are generated with sufficient intensity by the thread of the implant (even in a low-density pith), while the torque required for the blades’ action is low while maintaining a non-negligible self-aligning action.

#### 3.1.2. Definition of the Upper Rake Angle of the Main Cutting Edge, α

Conventionally, for a drill, the variables and cutting parameters are assumed to be localized at the outermost diameter of the drill, as they may vary depending on the position chosen for their characterization because of the typical morphology of the tool.

**Figure 10:**
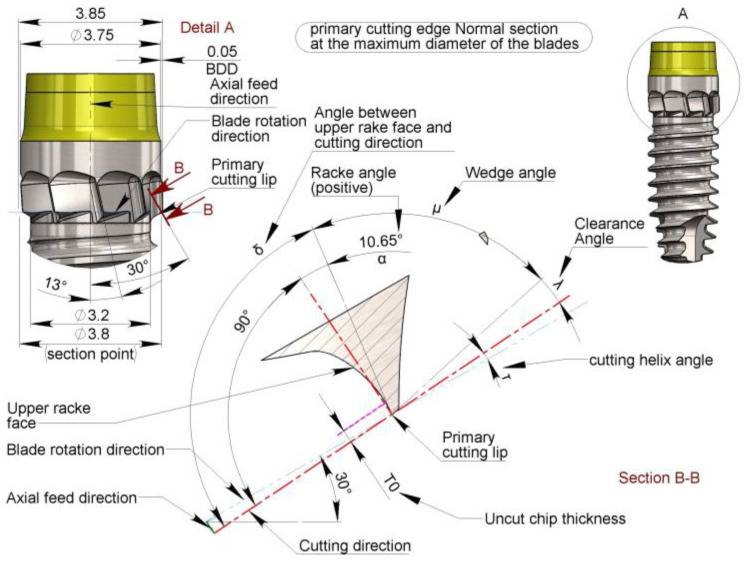
Normal section to the primary cutting edge at the maximum blade diameter. Note that α value results in a positive rake angle (δ > 90°). BDD: blade differential diameter.

The main cutting edge of the blades, derived from that of a traditional drill bit, has an upper face of the tool that forms an angle δ with respect to the cutting direction. With respect to the plane normal to the same cutting direction, the tool’s upper face forms an angle α, known as the top rake angle, which can be zero if it is perpendicular to the cutting direction vector (δ = 90°), positive if it forms an obtuse angle (δ > 90°), and negative if it forms an acute angle (δ < 90°) in respect to the cutting direction (Figure 10).

The roto translation of the implant during the screwing phase produces a penetration movement of the blades according to a precise inclination, called the working direction, corresponding to the helix angle of the implant thread.

The helix angle ψ, conventionally adopted for the external thread diameter d_1_, is determined by

(1) $$\psi =atan\left(\frac{p}{\pi \times d_{1}}\right)=atan\left(\frac{0.8\;mm}{\pi \times 3.8\;mm}\right)=3.834{}^{\circ}$$

However, the action diameter of blade d_3_ does not necessarily coincide with the external diameter of thread d_1_ because it depends on the Blade Differential Diameter (BDD) interference/play to be impressed according to the desired biodynamic requirements for the cortical bone.

If, for example, a differential is to be imposed on the blades BDD = +0.05 mm at a thread diameter d_1_ = 3.75 mm, the diameter of the blades d_3_ can be determined by the following formula:

(2) $$d_{3}=d_{1}+2\times \left(BDD\right)=3.75\;mm+2\times \left(+0.05\;mm\right)=3.85\;mm$$

The angle of the working direction $\;\psi_{1}$ adopting d_3_ instead of d_1_ would become

(3) $$\psi_{1}=atan\left(\frac{p}{\pi \times d_{3}}\right)=atan\left(\frac{0.8\;mm}{\pi \times 3.85\;mm}\right)=3.784{}^{\circ}$$

Because the blade diameter (d_3_) can be varied to best suit the biodynamic requirements of the cortical bone, given the negligible differences between ψ and ψ_1_, it is preferable to use d_1_, which is a constant of the implant, instead of d_4_, which is dependent on the BDD to be conferred.

Kalpakjian [41] stated that if the rake angle α is small, that is, it tends to 0, the chip tends to form in a discontinuous, fragmented type.

Top rake angles of 8° have been used in some studies [39,40]. However, both provide chip formation as a function of the depth of pass (UCP) and not as a function of the rake angle.

**Figure 11:**
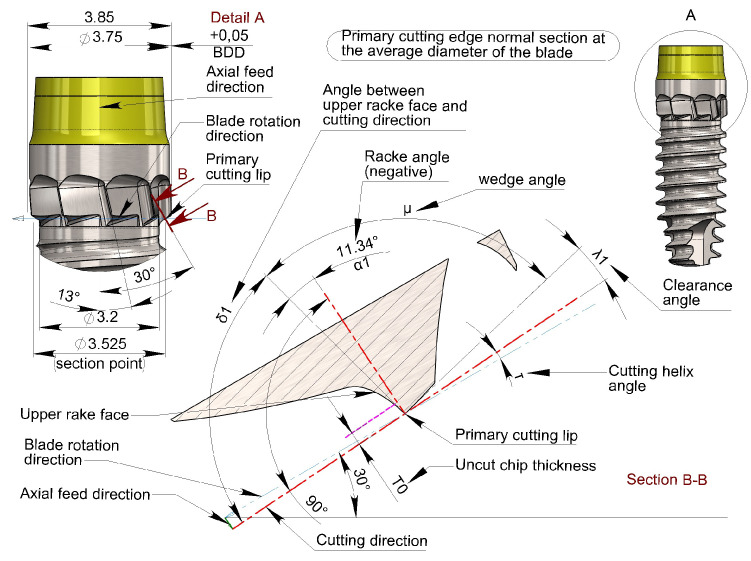
Normal section to the primary cutting edge at the average blade diameter. Note that α value results in a negative rake angle (δ < 90°).

In the blades, unlike conventional drill bits, the main cutting edge does not have a linear but a curved profile, owing to their construction method. Therefore, it is also necessary to consider the effect of blade construction geometry on the effective upper rake angle α. Starting from the outer diameter of the blade d_3_ toward the core diameter of the screw d_2_, the geometric effect involves the transformation of the upper rake angle α from positive (Figure 10) to negative (Figure 11).

To simplify the procedure for determining the upper rake angle α without committing major approximation errors, it is advisable to use the blade helix angle ε as a reference and then check that the ‘real’ rake angle α remains within a small geometric variation, arbitrarily set at values close to α = 0° +/− 10° in order to comply with the suggestion from other reports [39,40,41].

It follows that the helix angle of the blades ε, to obtain a rake angle above the main cutting edge, α = 0°, must be equal to ψ.

It is now necessary to consider that the helix angle ε of the blades also influences the extraction of the chip from the collecting cavity (and, therefore, from the osteotomy). If the helix angle is increased with respect to ψ, the filling of the collecting cavity tends to increase, conveying the chip toward the inside of the osteotomy. If it decreases, it tends to empty, driving the chip outward. If it coincides with ψ, it tends to leave the swarf at a random filling/ejection depending on the type of chips that the other operating conditions influence.

For the purpose of the invention, the bone chips should first fill the cavity, as this is supposed to promote the bone regeneration necessary for osseointegration [44,45,46], and only then achieve their complete filling, as the collecting cavities have a volume smaller than the excavated volume.

The excess bone chip must, therefore, be evacuated externally, avoiding producing much compression on the walls of the osteotomy, in accordance with the objective of the blades to reduce or control compression on the cortical bone. As a result, it is preferable to give an increased angle differential ε of +9° (arbitrarily chosen) to give a slightly forced harvesting effect because the type of continuous segmented chip (brittle), once the cavity is filled, is sufficiently “fluid” to be drained without significantly increasing the compressive effect on the bone.

As mentioned previously, the helix angle of the blades can be determined by

(4) $$\varepsilon =\psi +9{}^{\circ}=atan\left(\frac{p}{\pi \times d_{1}}\right)+9{}^{\circ}=atan\left(\frac{0.8\;mm}{\pi \times 3.75\;mm}\right)+9{}^{\circ}=12.834{}^{\circ}$$

This value can be approximated as an excess without appreciably affecting the desired effect at ε = 13°. The adoption of this angle results in variability (verified at CAD for simplicity) in the rake angle α:

$$\alpha =\left[\begin{array}{c} +10.65{}^{\circ}\\ -11.34{}^{\circ} \end{array}\right]$$

This value falls within the previously established limits to a reasonable degree; therefore, a helix angle of ε = 13° was chosen.

#### 3.1.3. Defining the Clearance Angle Below the Main Cutting Edge

**Figure 12:**
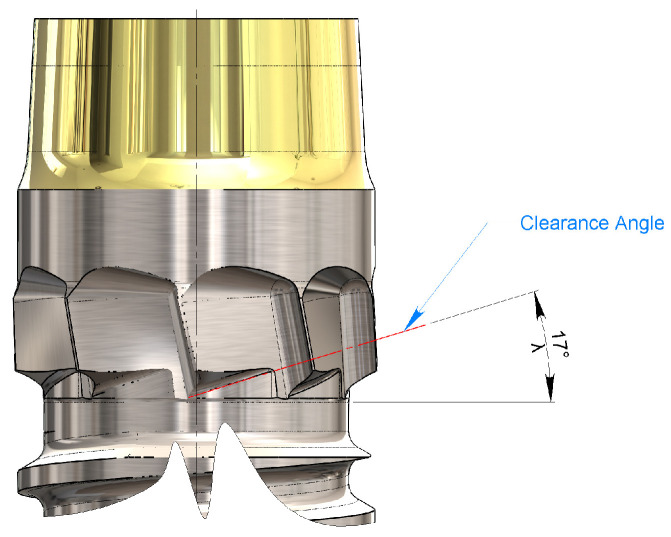
The clearance angle below the main cutting edge.

The clearance angle of the main cutting edge is denoted as λ, as shown in Figure 12. This allows the secondary flank of the cutting edge to not rub the freshly machined surface while compensating for the even minimal spring back that would push it against the tool. It has been suggested that λ should be between 12° and 15° for the cortical bone [47,48]. However, the axial feed pitch per revolution of the implant is very high (0.8 mm/revolution) compared to that of a normal drill (for the diameter in question, about 0.05 mm/revolution). The blades are integrated into the dental implant and, therefore, feed with the thread pitch. The helix angle ψ of the latter has already been defined in Equation (1) as ψ = 3.834°.

It is, therefore, necessary to increase the suggested λ-angle [47,48] (average value 13.5°) by an amount equal to the helix angle ψ of the thread to avoid rubbing the flank of the main cutting edge on the bone because of the high penetration pitch per revolution, which can be re-determined as

(5) $$\lambda =\psi +13.5{}^{\circ}=atan\left(\frac{p}{\pi \times d_{1}}\right)+13.5{}^{\circ}=atan\left(\frac{0.8\;mm}{\pi \times 3.8\;mm}\right)=17.334{}^{\circ}$$

This could be rounded to unity without incurring excessive approximations, that is, λ = 17°.

### 3.2. Parameters at the Secondary Cutting Edge

**Figure 13:**
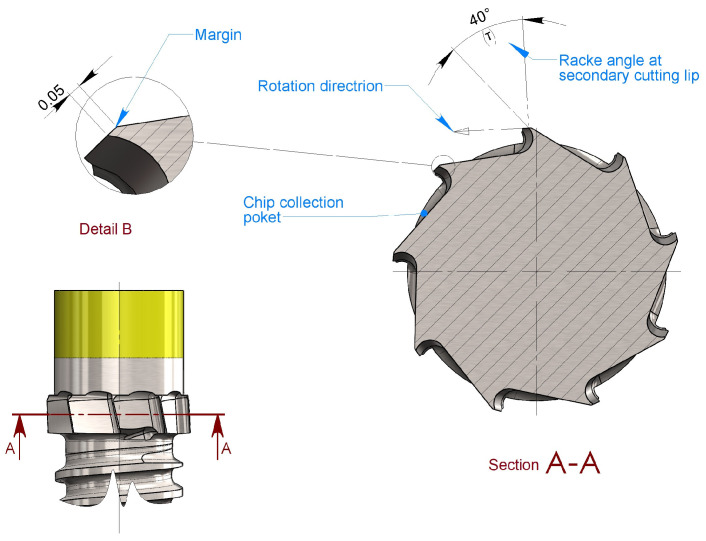
The secondary cutting edge: In section view A-A, the rake angle at the secondary cutting lip is denoted as τ. It does not show a clearance angle but has a slight cylindrical margin (detail B). This design creates a guiding surface to help maintain the implant centered within the osteotomy.

The secondary cutting edge (Figure 13) performs the function of scraping without further incising it, and the transcortical cylindrical surface is machined by the primary cutting edge, cleaning it, detaching any fragments remaining adhered to it, dragging them to the center of the flute of the blades to obtain the filling of the collection cavities, or, once the latter has been filled, guaranteeing their evacuation outside the osteotomy. To perform this function, an upper positive rake angle τ = 40° was arbitrarily chosen.

A clearance angle was not assigned to the secondary cutting edge. This is because the flank must perform the function of guiding the blades over the intra-cortical cylinder just created by the primary cutting edge. This means that this flank, also referred to as the blade edge, performs the function of restraining external loads that the operator might apply during screwing maneuvers, especially when using a hand ratchet.

The arbitrarily chosen margin geometry of 0.05 mm also helps to keep the friction torque low, which would otherwise increase the screwing torque of the implant, increasing the lateral forces to be applied and, consequently, potentially increasing the axial deflection.

### 3.3. Defining the Number of Blades

As in the drill bit, the main cutting edges are fed by the pitch p of the thread. Given the number of blades N_b,_ the axial feed per blade a can be determined from the following ratio:

(6) $$a=p/N_{b}$$

We must now consider that the blades have a vertex angle β, which, as mentioned above, influences the thickness of the chip being machined (UCT). The smaller the vertex angle β, the smaller the chip thickness UCT, according to the following mathematical relationship:

(7) $$UCT=a\times sin\left(\frac{\beta }{2}\right)$$

Substituting Equation (7) for Equation (6) and solving it for N_b_ yields the mathematical relationship that links the number of blades to the desired UCT chip thickness, vertex angle β, and thread pitch p:

(8) $$N_{b}=\frac{p\times sin\left(\frac{\beta }{2}\right)}{UCT}$$

To obtain the desired chip type (segmented continuous), a depth of cut (UCT) between 20 µm and 80 µm should be used [39]. The adoption of a UCT value at the lower limit, called UCT_0_, equal to 20 µm (UCT_0_ = 0.02 mm), produces a number of blades N_b_ equal to

(9) $$N_{b}=sin\frac{p\times cos\left(\frac{\beta }{2}\right)}{{UCT}_{0}}=\frac{0.8\;mm\times sin\left(\frac{120{}^{\circ}}{2}\right)}{0.020\;mm}=34.641≅35$$

A total of 35 blades were considered excessive because of the delicate construction form that would have to be given to them. In addition, the tools for making them are excessively fragile, and a high number of machining operations would result in high production costs.

The adoption of a depth of cut (UCT) value at the upper limit, called UCT_1_, equal to 80 µm (UCT_1_ = 0.08 mm), would produce a number of blades corresponding to

(10) $$N_{b}=\frac{p\times sin\left(\frac{\beta }{2}\right)}{{UCT}_{1}}=\frac{0.8\;mm\times sin\left(\frac{120{}^{\circ}}{2}\right)}{0.080\;mm}=8.660≅9$$

Nine blades would be acceptable from the perspective of the production economy. Furthermore, because the number of blades chosen is a multiple of the number of predetermined apical notches (N_i_ = 3), the latter could be banded with the blades, creating considerable advantages during implant processing.

By adopting N_b_ = 9 blades, the depth of cut actually applied (UCT_eff_) by a single blade can be re-determined as

(11) $${UCT}_{eff}=\frac{p\times sin\left(\frac{\beta }{2}\right)}{N_{b}}=\frac{0.8\;mm\times sin\left(\frac{120{}^{\circ}}{2}\right)}{9}=0.077\;mm$$

This value is within the set UCT limit for obtaining continuous segmented chips; therefore, a number of blades equal to N_b_ = 9 should be adopted.

## 4. Discussion

Almost all existing dental implants require a clinical protocol that involves thread interference with both medullary and cortical bone. This approach is used to provide the implant with maximum primary stability, even in cases of low-density bone.

Due to the design of most implants on the market, the greatest stability is achieved in the cortical layer through compression of the implant’s neck and threads against the bone. However, in the presence of a hard cortical layer, the implant can become stuck in the bone or cause fractures, particularly if the crest is thin.

On the other hand, in terms of primary stability, an excessively large osteotomy preparation, insufficient screwing torque, or weak bone resistance can lead to implant micromobility, especially with tapered implants. In these cases, the taper acts both as an amplifier of the thread-biting capability and as a potential accelerant for “unscrewing” if adequate boundary load conditions are not achieved (i.e., respecting bone deformations within its elastic field).

Therefore, it is essential to carefully control the “preload” applied to the dental implant during the screwing phase in an attempt to avoid excessive stress transfer from the bone–implant interface to the supporting bone, which could contribute to marginal bone loss [25,26,27,28,29,30,31].

Bone is a living tissue, and as such, even in implant surgery, it must be respected by maintaining its vitality as much as possible. One way to preserve this vitality is by avoiding excessive mechanical stress. The solution presented in this study could be key to controlling bone loss resulting from excessive mechanical stress, which is generally caused by the interference of the threads and neck of traditional dental implants with the cortical bone to achieve primary stability [20,49,50,51,52].

Recent animal studies have explored the performance and outcomes of implants featuring integrated blades, such as the Cortyblade^®^ system, which has potential clinical applications. In a study conducted on dogs, implants were placed in the edentulous alveolar ridge, and cortical blades with varying diameters were incorporated into the coronal portion of the implants to modify the preparation of the cortical bone crest [53]. The blade-to-collar diameter variations included −175 µm (designed to compress the marginal bone), 0 µm, +50 µm, and +200 µm, creating marginal gaps of corresponding sizes. Notably, this study demonstrated that implants with a 50 µm marginal gap exhibited the highest bone crest position, suggesting that this configuration may offer optimal conditions for bone preservation. The cortical blades showed favorable osseointegration, particularly in the +200 µm group, where collected bone particles were integrated into the newly formed bone. This highlights the potential for the blades to enhance both osseointegration and bone regeneration.

In another study utilizing rabbit tibiae, Cortyblade^®^ implants with blades measuring 0 µm, +50 µm, and +200 µm in diameter relative to the implant collar were investigated [54]. These blades allowed for precise shaping of the cortical bone and controlled decompression of the targeted areas. The results revealed successful osseointegration of both the implants and the blades, with effective preservation of the marginal bone and closure of marginal gaps ranging from 0 µm to 200 µm. These findings suggest that the use of implants with cortical blades can provide clinical advantages, particularly in terms of managing marginal bone levels and enhancing implant stability.

These animal model outcomes provide an understanding of the biomechanical behavior of Cortyblade^®^ implants, which can be translated into clinical practice. The variation in blade diameters presents an important topic for debate, as it raises questions about the ideal gap size for optimal osseointegration and bone preservation. This debate is crucial for guiding clinical decision-making and improving patient outcomes in implant dentistry.

In addition, as this new configuration of the implant is potentially able to right-size the implant socket since they are integrated into the implant, they could provide a further means of controlling the state of compression and/or decompression of the cortical layer, resulting from the unpredictable deviations imposed by the path of least resistance offered by the bone that opposes the pre-established implant positioning.

Furthermore, by collecting and forcing bone chips into the implant site, they can promote regeneration and osseointegration of the peri-implant tissues [53,54], potentially accelerating healing time and, thus, implant stabilization.

Blades eliminate radial cortical compression during implant insertion without interfering with the primary stability of the implant and promoting optimal osseointegration, as found in both studies cited [53,54].

With all the limitations of this study, the research establishes the basic principles for the proposed new solutions. These principles may represent a significant step toward optimizing the clinical protocol and predictability in relation to crestal bone loss.

## 5. Conclusions

The primary goal of this study was to develop an implant capable of achieving calibrated cortical bone preparation, ensuring both precise dimensional control and coaxial alignment. This design incorporates integrated blades that allow for controlled cortical decompression, helping to manage radial compressive stresses during implant placement.

Although the experimental studies cited were conducted independently of this research, they validate the functional efficacy of this implant design, demonstrating its ability to promote osseointegration and preserve marginal bone. The results suggest that this implant configuration holds the potential for improving clinical outcomes, particularly in cases where bone quality or density poses challenges to implant stability.

## 6. Patents

The innovations described in this article, relating to the CortyBlade^®^ dental implant, are covered by PCT No. PCT/IB2022/061392.

## Data Availability

Data are available upon reasonable request.
